# Risk factors for *Mycoplasma genitalium* infection among female sex workers: a cross-sectional study in two cities in southwest China

**DOI:** 10.1186/1471-2458-12-414

**Published:** 2012-06-07

**Authors:** Zhi Xiang, Yue-Ping Yin, Mei-Qin Shi, Ning Jiang, Yan Han, Hong-Chun Wang, Bing-Jie Zheng, Guo-Jun Liang, Xiang-Sheng Chen

**Affiliations:** 1 National Center for STD Control, Chinese Academy of Medical Sciences & Peking Union Medical College Institute of Dermatology, Nanjing, China

## Abstract

**Background:**

*Mycoplasma genitalium* (MG) is one of the common causes of non-gonococcal urethritis (NGU) in men and is associated with cervicitis, endometritis, and pelvic inflammatory diseases (PID) in women. The prevalence of MG infection has been reported to be high among female sex workers (FSWs) in many countries, but limited information is known among this population in China.

**Methods:**

From July to September 2009, venue-based FSWs were recruited in two cities (Wuzhou and Hezhou) of Guangxi Autonomous Region in southwest China. Information of socio-demographic and behavioral characteristics was collected by a questionnaire-based interview. Cervical specimens were obtained for detection of MG using a real-time polymerase chain reaction (PCR) assay targeting *mgpA* gene.

**Results:**

The overall prevalence of MG infection among 810 FSWs was 13.2% (95% CI = 10.87%–15.52%). MG infection was significantly associated with less education (adjusted odds ratio (AOR) = 2.36, 95% CI = 1.15–4.87) consisting of junior high school or below, being single (AOR = 2.27, 95% CI = 1.42–3.62), migrant background (AOR = 2.03, 95% CI = 1.29–3.20), and absence of any STI symptoms in the previous year (AOR = 1.66, 95% CI = 1.09–2.52).

**Conclusions:**

MG infection was prevalent among FSWs in the study areas. This pattern of infection suggests that an increasing attention should be paid to MG screening and treatment in this high risk population.

## Background

*Mycoplasma genitalium* (MG) was first isolated from the urethras of two men with nongonococcal urethritis (NGU) in 1980 [1], and it has been widely recognized as a sexually transmitted pathogen and one of the common causes of NGU [2]. MG is associated with cervicitis [3], endometritis [4], and pelvic inflammatory diseases (PID) [5] in women. If left untreated, MG infection can lead to tubal factor infertility [6,7].

In China, NGU has been resurgent since 1980s and used to be one of sexually transmitted infections (STIs) for case-reporting before 2008. Since then NGU has been replaced by *Chlamydia trachomatis* (CT) for national sentinel surveillance programme [8]. A few studies on MG were conducted in China, showing a prevalence rate of 20.6% to 25% among NGU patients [9,10], and 44.19% among non-chlamydial NGU patients [10]. These findings indicate that MG infection is prevalent in China. In women, MG is related to the inflammatory syndromes of the female reproductive tract. MG was found to be positively associated with urethritis and microscopic signs of cervicitis and/or mucopurulent cervical discharge in a literature review [11]. In addition, MG was found to be positively associated with PID [4,5][12,13]. The associations of MG with preterm birth and of MG with tubal infertility were also reported [6,7].

There have been many studies globally to determine the disease burden of MG and its associated factors in high-risk groups, particularly in female sex workers (FSWs). MG infection is common among FSWs. A study among FSWs in west Africa indicated that the prevalence of MG was higher than that of CT or *Neisseria gonorrhoeae* (NG) [14], and a prospective study showed that MG had a greater incidence (22.7 per 100 women-years) than that for both CT and NG among FSWs in Kenya [15]. In China, a few studies on MG prevalence in FSWs were conducted with relatively small sample size [16,17]. The limitation of such studies in China may be partially related to the ignorance of this infection in STD control programs and/or the lack of commercially available testing kits. The current study was aimed to determine the prevalence and the associated factors of MG infection among FSWs in two cities of Guangxi Autonomous Region in southwest China.

## Methods

### Study population

FSWs were recruited from varying work venues in two cities, Wuzhou and Hezhou, in Guangxi Autonomous Region, China. The venues where women solicited their sex clients in two study sites (Wuzhou and Hezhou) were mapped to determine the nature and location of each venue and a stratified random sample of the venues was identified. In each selected venue, a convenience sample of FSWs was recruited for questionnaire survey and cervical specimen collection. Eligibilities to participate in this study were females who were older than 18 years and reported providing commercial sex in the past year. The venues where FSWs were recruited included karaoke bars, star hotels, hair salons, barber shops, massage parlors, foot bathing shops, roadside shops, guesthouses, and roadside restaurants. FSWs who solicited on the streets or in other public outdoor places were recruited as well. The study protocol was reviewed and approved by the Ethics Committee of the National Center for STD Control in Nanjing, China.

### Survey methods

Eligible FSWs were invited to participate in the study after receiving a brief introduction of the study from outreach workers. After informed consent was obtained, the participants were interviewed by outreach workers with a structured questionnaire to collect information on socio-demographic characteristics, behavioral characteristics, and STI symptoms (discharge, pain, bleeding, discomfort, etc.) in the past year. Cervical swabs were collected by trained nurses.

### Laboratory test

Cervical swab samples were shipped to the National Reference Laboratory of the National Center for STD Control in Nanjing and stored at −70°C until testing for MG, The test was performed using a previously published and validated method of real-time PCR targeting the *mgpA* gene [18]. DNA was extracted from cervical swabs using the QIAmp DNA Mini Kit (Qiagen, GmbH, Germany) according to the manufacturer’s instructions. For detecting a 78 bp fragment of the *mgpA* gene, each PCR reaction was carried out in a total volume of 25 μl of reaction mixture containing 5 μl template DNA, 1× reaction buffer, 3.5 mM MgCl_2_, 200 μM dNTP mix, 0.625U HotStar Taq Polymerase (Qiagen, GmbH, Germany), 1.0 μM each primer (the forward primer MgPa-355 F, 5'-GAG AAA TAC CTT GAT GGT CAG CAA-3'; the reverse primer MgPa-432R, 5'-GTT AAT ATC ATA TAA AGC TCT ACC GTT GTT ATC-3'), and 0.1 μM MgPa-380 Taqman MGB probe (5'-FAM-ACT TTG CAA TCA GAA GGT-MGB-3') (Applied Biosystems, USA). PCR amplification was performed in a Lightcycler 480 instrument (Roche, Switzerland) under the following conditions: HotStar Taq Polymerase activation at 95°C for 10 min followed by a touch down protocol consisting of 1 cycle of denaturation at 95°C for 15 s, annealing at 64°C for 30 s, and extension at 72°C for 30 s; 1 cycle of denaturation at 95°C for 15 s, annealing at 62°C for 30 s, and extension at 72°C for 30 s; and 48 cycles of denaturation at 95°C for 15 s, annealing at 60°C for 30 s, and extension at 72°C for 30 s. We used a positive control in each batch of test by adding a known amount of MG to the PCR mixture as a control for the PCR efficiency.

### Data analysis

All data from the questionnaire were double entered into computer using EpiData software (EpiData Association, Denmark) by two research assistants. Comparisons of the categorical data were performed using Pearson χ2 test. The univariate regression analysis was used to preliminarily determine the risk factors associated with MG infection and estimate odds ratio (OR). The factors with significance level at p < 0.10 in the univariate analysis were included in a multivariate logistic regression model to the risk factors which independently associated with MG infection and estimate the adjusted odds ratio (AOR) as well as 95% confidence intervals (95%CI). A probability level of p < 0.05 was considered statistically significant. SPSS for Windows 13.0 (SPSS Inc, USA) was used for statistical analysis.

## Results

### Socio-Demographic characteristics

From July to September 2009, a total of 810 FSWs were recruited from the two cities. The mean age was 26.53 years old (range 18–52 years old), and 80.6% (652/809) of them were of Han ethnicity. Out of the 810 participants, 15.8% received an education at senior high school or above, 57.4% were single. Less than half (41.6%, 336/808) of the participating FSWs were local registered residents. Similar numbers of FSWs were recruited in Wuzhou City (406) and Hezhou City (404).

### STI symptoms and sexual behaviors

Three-quarters (598/810) of the participants received at least one of the following kinds of health care service in the previous year: the introduction of STI and AIDS prevention knowledge, STI examination or treatment, testing and consulting for HIV and syphilis, the introduction of condom use, and free condoms. Among 810 participants, 57.4% (465/810) presented at least one of the STI symptoms (discharge, pain, bleeding, discomfort, etc.) in the previous year, including 37.5% who had only one symptom and 19.9% who had more than one. Majority (82.1%, 665/810) of participants reported condom use in the most recent sexual transaction. Over half (59.6%) reported consistent condom use, 31.1% reported used condoms sometimes during the previous month, and 5.3% reported never using a condom. Of 807 participants, 11.8% (95/807) reported drug use in the previous year.

### *Mycoplasma genitalium* prevalence and risk factors

The real-time PCR successfully amplified the 78 bp fragment of the *mgpA* gene in 107 of 810 cervical swabs, yielding the overall MG prevalence of 13.2% (95% CI = 10.87%–15.52%).

In the univariate analysis, MG infection was significantly associated with less education consisting of junior high school and below, being single, living alone or with partners rather than husbands, migrant background, and absence of STI symptoms in the previous year. No association was observed between MG infection and ethnicity, health care in the previous year, condom use in the most recent sexual transaction, frequency of condom use in the previous month and study city.

Multivariate analysis indicated that MG infection was only independently associated with less education (AOR = 2.36, 95% CI = 1.15–4.87), being single (AOR = 2.27, 95% CI = 1.42–3.62), migrant backgrounds (AOR = 2.03, 95% CI = 1.29–3.20), and absence of STI symptoms in the previous year (AOR = 1.66, 95% CI = 1.09–2.52). Socio-demographic and behavioral characteristics, univariate and multivariate analysis results are shown in Table 1.

**Table 1:** **Socio-demographic and behavioral characteristics, and factors associated with *Mycoplasma genitalium *infection among female sex workers in Guangxi, China**

**Variables**	**Female sex workers**	**MG infection**	**OR^1^ (95% CI^2^)**	**AOR^3^(95% CI^2^)**
	**Number (%)**	**Number (%)**		
Age (year)			p = 0.38	
≤20	152(18.8)	24(15.8)	1.63 (0.87-3.06)	
21-25	276(34.1)	35(12.7)	1.27 (0.71-2.25)	
26-30	177(21.9)	27(15.3)	1.57 (0.85-2.89)	
≥ 31	204(25.2)	21(10.3)	1	
Ethnicity			p = 0.1	
Han	652(80.6)	80(12.3)	1	
Ethnic minorities	157(19.4)	27(17.2)	1.48 (0.92-2.39)	
Education			p = 0.028	p = 0.02
Junior high school or below	682(84.1)	98(14.4)	2.22 (1.09-4.52)	2.36 (1.15-4.87)
Senior high school or above	128(15.8)	9(7.0)	1	1
Marital status			p < 0.001	p < 0.001
Currently married	317(39.1)	28(8.8)	1	1
Single	465(57.4)	78(16.8)	2.08 (1.32-3.29)	2.27 (1.42-3.62)
Divorce	28 (3.5)	1(3.6)	0.38 (0.05-2.92)	0.37 (0.05-2.87)
Cohabiting status			p = 0.012	
Living with spouse	134(16.6)	7(5.2)	1	
Living alone	321(39.7)	43(13.4)	2.81 (1.23-6.41)	
Living with others	354 (43.6)	56(15.8)	3.41 (1.51-7.69)	
Residential status			p = 0.005	p = 0.002
Local residents	336(41.6)	31(9.2)	1	1
Not local	472(58.4)	76(16.1)	1.89 (1.21-2.94)	2.03 (1.29-3.20)
City of recruitment			p = 0.37	
Wuzhou	406(50.2)	58(14.3)	1.20 (0.80-1.81)	
Hezhou	404(49.8)	49(12.1)	1	
Receiving health care in the past year			p = 0.63	
Yes	598(73.8)	77(12.9)	1	
No	212(26.2)	30(14.2)	0.90 (0.57-1.41)	
Presenting STI symptoms in the past year			p = 0.03	p = 0.02
Yes	465(57.4)	51(11.0)	1	1
No	345(42.6)	56(16.2)	1.57 (1.05-2.37)	1.66 (1.09-2.52)
Condom use during last transaction			p = 0.34	
No	119(14.7)	14(11.8)	1	
Yes	665(82.1)	92(13.8)	1.20 (0.67-2.19)	
Declined to answer	26(3.2)	1(3.8)	0.3 0 (0.04-2.39)	
Condom use frequency with clients in the past month			p = 0.313	
Never	43(5.3)	5(11.6)	1	
Every time	483(59.6)	62(12.8)	1.12 (0.42-2.95)	
Sometimes	252(31.1)	39(15.5)	1.39 (0.52-3.76)	
Declined to answer	32(4.0)	1(3.1)	0.25 (0.03-2.21)	
Drug use in the past year			p = 0.062	
No	712(87.8.)	88(12.4)	1	
Yes	99(12.2)	19(19.2)	1.68 (0.97-2.91)	

^1^Odds Ratio.

^2^95% confidence interval.

^3^ Adjusted Odds Ratio.

## Discussion

In this study, the MG prevalence of 13% based on PCR assay among 810 FSWs in two cities in southwest China is similar to that reported among FSWs in other Asian countries (12% in Indonesia [19] and 12% in Japan [20]), but lower than that in some African countries with high HIV prevalence (26%in west Africa [14] and 16% in Kenya [15]). In comparison with other areas in China, this rate is slightly lower than that 16% and 17% reported in two studies with small sample sizes (72 and 75) of FSWs in Jiangsu Province in 2005 [16,17]. A literature review based on 48 published reports of MG urogenital infection in high- or low-risk populations worldwide indicates an overall prevalence of 7% and 2%, respectively [11]. The prevalence of MG in our study population is higher than not only the overall prevalence in low-risk but also that in high-risk population, indicating the needs for public health interventions in the study areas.

In the present study, we assessed the associations between MG infection and socio-demographic and behavioral characteristics and STI symptoms (discharge, pain, bleeding, discomfort, etc.). A significant association between MG infection with less education is consistent with the finding of an earlier study in west African [14]. This association might be attributed to working on street and outdoor places with more sexual partners, and less knowledge with STI prevention, and poor seeking health care services of those FSWs with less education. Single FSWs had higher MG prevalence, and single status is related to younger age. More sexual activity, and possibly less knowledge and experience with STI prevention, including less use of a condom [21], could be reflected by younger age.

Unlike prior cross sectional studies of women [14,20], absence of STI symptoms in the past year was associated with MG infection in this study, The lack of association between MG infection and the presence of STI symptoms might be due to the following reasons. First, presence of STI symptoms in the past year were self-reported and recall bias might exist. The subjective feeling and limited nature of reproductive tract symptoms also could contribute to the causal associations with MG infection. Second, if STI symptoms were very slight, they could be ignored. Third, the biologically confirmed rate of current sexually transmitted infection has been repeatedly found to be higher than self-reported STI history [22]. For instance, in one study, only 19.4% of 454 FSWs in Guangxi reported a history of STI, while 41.5% of them tested positive for at least one STI [23]. Furthermore, younger women may have more biological susceptibility to some STIs due to cervical ectopy and less likelihood of acquired immunity from previous STI exposure [24,25]. This may be a reason why a past history of STIs was negatively associated with prevalent STI infection in this study. However, further studies are needed to provide more evidence on the protective immunity related to MG infection.

The finding of our study suggests that MG infection in women is more likely to be asymptomatic or have few slight symptoms, which may easily be ignored and undiagnosed.

In China, rural-to-urban migrants have been found to have higher sexual risks than local residents [26]. Migrant FSWs have been found to have low rates of condom use and high rates of STIs in many studies [27,28]. This risk behavioral characteristics of migrant FSWs is also reflected by a higher prevalence of MG observed in our study. The association between condom use and MG infection was not observed in this study, which was consistent with the results from the west African FSWs study as well as the Uganda FSWs study [14,29].

Even though the overall HIV prevalence in China is low, HIV infection through heterosexual transmission is increasing [30]. In light of the rapid spread of classic STDs with high prevalence in FSWs [31] and the extensive use of commercial sex by their clients, FSWs are a high risk population for HIV/STD infection and for increasing the risk of HIV transmission. A significant association between MG and HIV infection has been observed in many cross-sectional studies and is well-reviewed. In a systematic review and meta-analysis of 19 studies, MG infection was associated with a 2-fold increased risk of HIV infection [32]. MG organism burden is associated with the shedding of HIV-1 DNA from the cervix [33], suggesting that MG infection may facilitate HIV transmission. Regarding the high prevalence of MG infection among FSWs in China, active interventions of MG infection will have not only implications for decreasing disease burden caused by MG but also preventing sexual transmission of HIV among this population and their clients.

After the initial isolation of MG three decades ago, it has been repeatedly identified as a STI pathogen. Although MG prevalence in high-risk population such as FSWs is gaining increasing attention worldwide, only a few studies estimated MG prevalence among small Chinese FSW populations. This report is the first to determine the prevalence and risk factors of MG among a large Chinese FSW population.

Since MG is extremely difficult to culture, several PCR methods are now commonly used to tested MG, including: conventional 16 S rRNA gene PCR, real-time 16 S rRNA gene PCR and real-time *mgpA* gene PCR. Jenson and his research team developed the real-time *mgpA* gene PCR method in 2004, they also compared the specificity and sensitivity of this method with conventional PCR for detection of the 16 S rRNA gene. Results showed real-time *mgpA* gene PCR to be more sensitive and have less inhibition [18]. Another subsequent study compared the above three different PCR assays, and results showed that among them, real-time *mgpA* gene PCR had the highest sensitivity. The authors concluded that it was well suited for the clinical diagnosis of MG [34]. Thus, we chose real-time *mgpA* gene PCR to test for MG in our study.

The present study has several limitations. First, data of socio-demographic and behavioral characteristics were self-reported from face-to-face interview, recall and report bias can be a concern. Second, as our study subjects were not a representative sample of the FSWs population and a convenience sample based on venue-based recruitment was used, any generalization of the results from this study should therefore be made with caution. Third, when we compare the MG prevalence among studies, we should keep in mind that due to the use of different test methods, sensitivity and specificity may vary and therefore may partly affect the results. Since we did not perform the inhibition analysis by using the real-time *mgpA* gene PCR assay, MG infection rate may be higher and accuracy of the test results might also be influenced by the quality of the samples. Finally, this study did not investigate the associations of HIV and other STIs with MG infection.

## Conclusions

In this study, the overall prevalence of MG infection was found to be substantial among FSWs in study areas. These findings highlight the needs for increased attention towards prevention and intervention of MG infection among FSWs. Routine screening, timely diagnosis, and treatment of MG infection should be considered in comprehensive HIV/STI control program in China.

## Competing interests

The authors declare that they have no competing interests.

## Authors’ contributions

ZX, YPY and XSC designed the study and wrote the initial manuscript. NJ and GJL assisted the field work and data collection. MQS, YH, HCW, ZX and BJZ did the laboratory test. XZ did the data analysis. XSC edited the manuscript and completed the final revisions. All authors read and approved the final manuscript.

## Pre-publication history

The pre-publication history for this paper can be accessed here:

http://www.biomedcentral.com/1471-2458/12/414/prepub

## References

[B1] Tully JG Taylor-Robinson D Cole RM Rose DL A newly discovered mycoplasma in the human urogenital tract Lancet 1981 1 1288 1291 6112607 10.1016/s0140-6736(81)92461-2

[B2] Gaydos C Maldeis NE Hardick A Hardick J Quinn TC *Mycoplasma genitalium* compared to chlamydia, gonorrhoea and trichomonas as an aetiological agent of urethritis in men attending STD clinics Sex Transm Infect 2009 85 438 440 10.1136/sti.2008.035477 19383597 PMC2761964

[B3] Falk L Fredlund H Jensen JS Signs and symptoms of urethritis and cervicitis among women with or without *Mycoplasma genitalium or Chlamydia trachomatis* infection Sex Transm Infect 2005 81 73 78 10.1136/sti.2004.010439 15681728 PMC1763725

[B4] Cohen CR Manhart LE Bukusi EA Astete S Brunham RC Holmes KK Sinei SK Bwayo JJ Totten PA Association between *Mycoplasma genitalium* and acute endometritis Lancet 2002 359 765 766 10.1016/S0140-6736(02)07848-0 11888591

[B5] Simms I Eastick K Mallinson H Thomas K Gokhale R Hay P Herring A Rogers PA Associations between *Mycoplasma genitalium, Chlamydia trachomatis* and pelvic inflammatory disease J Clin Pathol 2003 56 616 618 10.1136/jcp.56.8.616 12890814 PMC1770020

[B6] Clausen HF Fedder J Drasbek M Nielsen PK Toft B Ingerslev HJ Birkelund S Christiansen G Serological investigation of *Mycoplasma genitalium* in infertile women Hum Reprod 2001 16 1866 1874 10.1093/humrep/16.9.1866 11527890

[B7] Svenstrup HF Fedder J Kristoffersen SE Trolle B Birkelund S Christiansen G *Mycoplasma genitalium, Chlamydia trachomatis*, and tubal factor infertility–a prospective study. Fertil Steril 2008 90 513 520 10.1016/j.fertnstert.2006.12.056 17548070

[B8] National Center for STD Control, CDC, China http://www.ncstdc.org/yqjc-detail/work/work_008.pdf

[B9] Wang HY Shi MQ Ye SZ Lai WH Zhang CF Jiang WH Xue HZ Wang HC *Mycoplasma genitalium* infection in 451 patients with sexually transmitted diseases J Clin Dermatol 2002 31 543 544

[B10] Jiangjuan Study on the Association of Mycoplasma Genitalium and Ureaplasma Urealyticum in Men with acute Nongonococcal Urethritis 2004 PhD thesis. Peking Union Medical College Institute of Dermatology

[B11] McGowin CL Anderson-Smits C *Mycoplasma genitalium*: an emerging cause of sexually transmitted disease in women PLoS Pathog 2011 7 e1001324 10.1371/journal.ppat.1001324 21637847 PMC3102684

[B12] Bjartling C Osser S Persson K The association between *Mycoplasma genitalium* and pelvic inflammatory disease after termination of pregnancy BJOG 2010 117 361 4 10.1111/j.1471-0528.2009.02455.x 20015303

[B13] Haggerty CL Totten PA Astete SG Lee S Hoferka SL Kelsey SF Ness RB Failure of cefoxitin and doxycycline to eradicate endometrial *Mycoplasma genitalium* and the consequence for clinical cure of pelvic inflammatory disease Sex Transm Infect 2008 84 338 42 Epub 2008 Apr 29 10.1136/sti.2008.030486 18445635 PMC2572206

[B14] Pépin J Labbé AC Khonde N Deslandes S Alary M Dzokoto A Asamoah-Adu C Méda H Frost E *Mycoplasma genitalium*: an organism commonly associated with cervicitis among west African sex workers Sex Transm Infec 2005 81 67 72 10.1136/sti.2003.009100 15681727 PMC1763741

[B15] Cohen CR Nosek M Meier A Astete SG Iverson-Cabral S Mugo NR Totten PA *Mycoplasma genitalium* infection and persistence in a cohort of female sex workers in Nairobi, Kenya Sex Transm Dis 2007 34 274 279 16940898 10.1097/01.olq.0000237860.61298.54

[B16] Pingmin W Yuepu P Jiwen Z Prevalence survey on condom use and infection of urogenital mycoplasmas in female sex workers in China Contraception 2005 72 217 220 10.1016/j.contraception.2005.05.002 16102559

[B17] Wei PH Pu YP Zhao JW Nested PCR for detection of seven pathogenic mycoplasma species from prostitutes and patients with genital infection J Environ Occup Med 2005 22 232 235

[B18] Jensen JS Björnelius E Dohn B Lidbrink P Use of TaqMan 5' nuclease real-time PCR for quantitative detection of *Mycoplasma* genitalium DNA in males with and without urethritis who were attendees at a sexually transmitted disease clinic J Clin Microbiol 2004 42 683 692 10.1128/JCM.42.2.683-692.2004 14766837 PMC344445

[B19] Mawu FO Davies SC McKechnie M Sedyaningsih ER Widihastuti A Hillman RJ Sexually transmissible infections among female sex workers in Manado, Indonesia, using a multiplex polymerase chain reaction-based reverse line blot assay Sex Health 2011 8 52 60 10.1071/SH10023 21371382

[B20] Tsunoe H Tanaka M Nakayama H Sano M Nakamura G Shin T Kanayama A Kobayashi I Mochida O Kumazawa J Naito S High prevalence of *Chlamydia trachomatis, Neisseria gonorrhoeae* and *Mycoplasma genitalium* in female commercial sex workers in Japan Int J STD AIDS 2000 11 790 794 10.1258/0956462001915291 11138913

[B21] Wang B Li X Stanton B Zhang L Fang X Alcohol use, unprotected sex, and sexually transmitted infections among female sex workers in China Sex Transm Dis 2010 37 629 636 20601927 10.1097/OLQ.0b013e3181e2118a PMC2943995

[B22] Hong Y Li X Behavioral studies of female sex workers in China: a literature review and recommendation for future research AIDS Behav 2008 12 623 636 10.1007/s10461-007-9287-7 17694431

[B23] Wang B Li X Stanton B Yang H Fang X Zhao R Dong B Zhou Y Liu W Liang S Vaginal douching, condom use, and sexually transmitted infections among Chinese female sex workers Sex Transm Dis 2005 32 696 702 10.1097/01.olq.0000175403.68410.ec 16254545 PMC1935451

[B24] Pettifor AE Turner AN Van Damme K Hatzell-Hoke T Rasamindrakotroka A Nasution MD Behets F Increased risk of chlamydial and gonococcal infection in adolescent sex workers in Madagascar Sex Transm Dis 2007 34 475 478 17237736 10.1097/01.olq.0000251239.85295.81

[B25] Rekart ML Brunham RC Epidemiology of chlamydial infection: are we losing ground? Sex Transm Infect 2008 84 87 91 10.1136/sti.2007.027938 18216155

[B26] Liu H Li X Stanton B Liu H Liang G Chen X Yang H Hong Y Risk factors for sexually transmitted disease among rural-to-urban migrants in China: implications for HIV/sexually transmitted disease prevention AIDS Patient Care STDS 2005 19 49 57 10.1089/apc.2005.19.49 15665635 PMC1935434

[B27] Bautista CT Mosquera C Serra M Gianella A Avila MM Laguna-Torres V Carr JK Montano SM Sanchez JL Immigration status and HIV-risk related behaviors among female sex workers in South America AIDS Behav 2008 12 195 201 10.1007/s10461-007-9270-3 17587171

[B28] del Amo J González C Losana J Clavo P Muñoz L Ballesteros J García-Saiz A Belza MJ Ortiz M Menéndez B del Romero J Bolumar F Influence of age and geographical origin in the prevalence of high risk human papillomavirus in migrant female sex workers in Spain Sex Transm Infect 2005 81 79 84 10.1136/sti.2003.008060 15681729 PMC1763723

[B29] Vandepitte J Muller E Bukenya J Nakubulwa S Kyakuwa N Buvé A Weiss H Hayes R Grosskurth H Prevalence and correlates of *Mycoplasma genitalium* infection among female sex workers in Kampala, Uganda J Infect Dis 2012 205 289 296 10.1093/infdis/jir733 22102734

[B30] Wang L Wang N Wang L Li D Jia M Gao X Qu S Qin Q Wang Y Smith K The 2007 Estimates for people at risk for and living with HIV in China: progress and challenges. J Acquir Immune Defic Syndr 2009 50 414 418 10.1097/QAI.0b013e3181958530 19214116

[B31] van den Hoek A Yuliang F Dukers NH Zhiheng C Jiangting F Lina Z Xiuxing Z High prevalence of syphilis and other sexually transmitted diseases among sex workers in China: potential for fast spread of HIV AIDS 2001 15 753 759 10.1097/00002030-200104130-00011 11371690

[B32] Napierala Mavedzenge S Weiss HA Association of *Mycoplasma genitalium* and HIV infection: a systematic review and meta-analysis AIDS 2009 23 611 620 10.1097/QAD.0b013e328323da3e 19194271

[B33] Manhart LE Mostad SB Baeten JM Astete SG Mandaliya K Totten PA High *Mycoplasma genitalium* organism burden is associated with shedding of HIV-1 DNA from the cervix J Infect Dis 2008 197 733 736 10.1086/526501 18266605 PMC4090222

[B34] Edberg A Jurstrand M Johansson E Wikander E Höög A Ahlqvist T Falk L Jensen JS Fredlund H A comparative study of three different PCR assays for detection of *Mycoplasma genitalium* in urogenital specimens from men and women. J Med Microbiol 2008 57 304 309 10.1099/jmm.0.47498-0 18287292

